# A Review: Epigenetic Mechanism in Ochratoxin A Toxicity Studies

**DOI:** 10.3390/toxins9040113

**Published:** 2017-03-23

**Authors:** Liye Zhu, Boyang Zhang, Yaqi Dai, Hongyu Li, Wentao Xu

**Affiliations:** 1 Beijing Advanced Innovation Center for Food Nutrition and Human Health, College of Food Science & Nutritional Engineering, China Agricultural University, Beijing 100083, China; zlyhome@163.com (L.Z.); boyang0214@126.com (B.Z.); 2 Beijing Laboratory for Food Quality and Safety, College of Food Science and Nutritional Engineering, China Agricultural University, Beijing 100083, China; DYQ5230@163.com (Y.D.); 18801061867@163.com (H.L.); 3 The Supervision, Inspection and Testing Center of Genetically Modified Organisms, Ministry of Agriculture, Beijing 100083, China

**Keywords:** Ochratoxin A, epigenetic mechanism, DNA methylation, non-coding RNA, histone modification

## Abstract

Ochratoxin A (OTA) is a natural contaminant that has displayed nephrotoxicity and hepatotoxicity in mammals. It contaminates a great variety of foodstuffs and threatens people’s lives. The molecular mechanism of OTA-induced toxicity has been studied since 1965. Moreover, epigenetic mechanisms are also studied in OTA-induced toxicity. Additionally, the mode of OTA epigenetic research has been advanced in research hotspots. However, there is still no epigenetic study of OTA-induced toxicity. In this review, we discuss the relationship between these epigenetic mechanisms and OTA-induced toxicity. We found that studies on the epigenetic mechanisms of OTA-induced toxicity all chose the whole kidney or liver as the model, which cannot reveal the real change in DNA methylation or miRNAs or histone in the target sites of OTA. Our recommendations are as follows: (1) the specific target site of OTA should be detected by advanced technologies; and (2) competing endogenous RNAs (ceRNA) should be explored with OTA.

## 1. Introduction of Mycotoxins and Ochratoxin A

### 1.1. Mycotoxins

Mycotoxins are secondary metabolites produced by fungi, including *Penicillium*, *Aspergillus*, *Fusarium* and *Alternaria* [1]. These mycotoxins always contaminate foodstuffs and cereals, and they have become one of the larger concerns in human health. They can produce nephrotoxic, hepatotoxic, carcinogenic, mutagenic, teratogenic and immunosuppressive effects. Aflatoxin (AFB), ochratoxin (OTA), zearalenone (ZEA), deoxynivalenol (DON), fumonisim B_1_ (FB_1_) and T_2_ toxin are the main contaminants that have been researched extensively [2].

To safeguard the health of humans and farm animals, many counties worldwide have established regulations on the contents of mycotoxins in food and feed. The regulations of the contents of mycotoxins are different every country. They are based on two main reasons: (1) different optimal environmental conditions of mycotoxin production; (2) different modes of action (MOA) of mycotoxins. To provide more precise toxicity assessment, research on the molecular mechanism of mycotoxins has been explored in recent years. These studies have mainly focused on oxidative stress, cell apoptosis, cell autophagy and DNA adduct formation.

In 1955, the first conference on food additives was held by the FAO/WHO. Since then, mycotoxins have been a source of concern. Aflatoxins were first evaluated in 1987, but the tolerable intake level was not established because the levels of contamination in foodstuffs vary widely worldwide. Aflatoxins B, G, and M were evaluated in detail (WHO, 1998). OTA was re-evaluated in 1995 [3]. Among these, the carcinogenic potency of AFB_1_ has been evaluated and verified. However, until now, there has been no definitive conclusion concerning the carcinogenic potency of OTA.

### 1.2. Ochratoxin A

OTA was initially described in 1965 [4]. It is produced mainly by *Aspergillus ochraceus* and *Penicillum verrucosum* and is a pentaketide derived from a dihydrocoumarin family derivative coupled to β-phenylalanine. OTA is distributed throughout the world, especially in the Balkans. In this area, a chronic renal disease, Balkan endemic nephropathy (BEN), is found. After an investigation concerning the etiology of BEN, OTA was suspected because of the more frequent OTA contamination [5]. In subsequent studies, more toxicity was found to be induced by OTA, including nephrotoxicity, hepatotoxicity, teratogenicity, immunotoxicity, neurotoxicity and genotoxicity [6]. Moreover, the IARC (International Agency for Research on Cancer) has classified OTA as a Group 2B carcinogen. More studies should focus on the molecular mechanisms of OTA-induced toxicity.

OTA contaminates a great variety of foodstuffs, such as grapes, coffee, cocoa, and infant food. It is of great concern for human health. International organizations worldwide have always tried to establish the tolerable dose of OTA. However, due to inconsistent evaluation standards, the limit of the OTA dose is different. In 1991, the Nordic Expert Group on Food Safety set the tolerable daily intake (TDI) at 5 ng/kg·bw/day. In 2006, the European Food Authority (EFSA) set the provisional tolerable weekly intake (PTWI) at 120 ng/kg·bw/week. Additionally, the Scientific Committee of Food (SCF) of the European Union set the provisional tolerable daily intake (PTDI) at 5 ng/kg·bw/day. In 2007, the Joint FAO/WHO Expert Committee on Food Additives (JECFA) reset the PTWI down to 100 ng/kg·bw/week. However, in 2010, Health Canada set the PTDI and negligible cancer risk intake (NCRI) at 3 and 4 ng/kg·bw/day [7]. Based on further study of the MOA of OTA, a more precise evaluation will be established.

## 2. Main Mechanisms of Ochratoxin A–Induced Toxicity

### 2.1. Oxidative Stress

Oxidative stress has been recognized as a potential factor related to some diseases. The adverse effects induced by oxidative stress are DNA damage [8], protein damage [9] and lipid damage [10]. Most researchers have reported that oxidative stress is one of the MOAs of OTA. In vitro, OTA increases the concentrations of malondialdehyde and lipid peroxides in kidney cells [11]. OTA is accumulated in the proximal tubule in the kidney. OTA exposure of primary rat PT cells and LLC-PK1 cells resulted in a concentration-dependent elevation of the reactive oxygen species (ROS) level, a depletion of cellular glutathione (GSH) levels and an increase in the formation of 8-oxoguanine. Moreover, cellular GSH levels play a pivotal role in limiting the short-term toxicity of this mycotoxin in renal tubular cells [12]. In HepG2 cells and Vero cells, OTA induced the production of ROS, and decreased the SOD activity [13,14]. In vivo, OTA-induced oxidative stress is found in the liver. In 2001, compared with the control group, the level of lipid peroxide (LPO) in the liver was significantly increased after OTA treatment. Concomitantly, the levels of GSH and the enzyme activities of SOD, CAT, GR and glutathione peroxidase (GSPx) in the liver were significantly decreased [15]. OTA is an *Nrf2* inhibitor. OTA can induce the inhibition of *Nrf2* activation and *Nrf2* gene transcription. *Nrf2* inhibition is the primary mechanism of OTA’s toxicity. Thereafter, it can induce the increase in ROS, subsequently leading to lipid peroxidation, proteotoxic stress and oxidative DNA damage [16]. The OTA-induced decrease of HO-1 may also explain the induction of oxidative stress [17].

### 2.2. Cell Apoptosis

More and more researchers have recognized that OTA can induce cell apoptosis. OTA can induce apoptosis in vivo and in vitro. In vivo, apoptosis was found in the kidneys of mice and rats [18,19]. It was also found in vitro, including in human HeLa cells [19], HepG2 cells [20], HEK293 cells, V79 cells, CV-1 cells, PRK cells [8], PK15 cells [21], and MDCK-C7 cells [22]. This apoptosis is activated by various signal transduction pathways. Among these pathways, the ERK1/2 pathway [23] and the JNK/SAPK pathway are the major ones [24]. It was reported that their involvement resulted in fragments in OTA-induced cell death. The MAPK/ERK pathway can convey a signal from a receptor on the cell surface to the nuclear DNA. Additionally, MAP3Ks, which are MAP kinase kinases, comprise the three-tiered cascade of MAPK activation. ASK is a MAP3K family member. ASK1 participated in the OTA-induced inhibition of mRNA splicing, nucleotide metabolism, cell cycle arrest, DNA repair inhibition, and the activation of lipid metabolism [25]. ASK1 played an essential role in promoting OTA-induced renal cytotoxicity. All these data proved that cell apoptosis is one of the patterns of OTA-induced cytotoxicity.

### 2.3. Cell Autophagy

Cell autophagy is an adaptive response to stress in diseases. The change in the microenvironment will influence the role of autophagy in cellular processes. Autophagy and mitophagy, in principle, serve an adaptive role to protect organisms against diverse pathologies. Additionally, the function of autophagy and mitophagy in OTA-induced toxicity has been explored. Mitochondrial dysfunction is an early event during the development of OTA toxicity [26,27], while Nix functions as a regulated mitophagy receptor [28]. In 2014, Shen et al. found that OTA induced autophagy and mitophagy. In Nix-deficient HEK293 cells, cell death was more severe after OTA treatment. Moreover, the pro-apoptotic Bad and AIF proteins were up-regulated by OTA. These results revealed that Nix plays a central role in autophagy and mitophagy, protecting cells against OTA-induced renal toxicity.

### 2.4. Calcium Homeostasis

The disruption of calcium homeostasis, leading to a sustained increase in the cytosolic calcium level, has been associated with cytotoxicity in response to various agents in different cell types [29]. Both in vivo and in vitro, the effect of OTA on calcium homeostasis was studied. In 1989, research revealed that OTA can inhibit the rate of ATP-dependent calcium uptake by 42%–45%. Moreover, they found that the disruption of calcium homeostasis induced by OTA is due to an impairment of the endoplasmic reticulum membrane, probably via enhanced lipid peroxidation [30]. In the rat kidney, OTA administered to rats resulted in an increase in renal endoplasmic reticulum calcium pump activity [29]. OTA could also cause the modulation of the intracellular calcium level in Syrian hamster embryo (SHE) fibroblasts. Additionally, the modulation further induced the disruption of mitotic disturbances, resulting in cytotoxicity [31].

### 2.5. DNA Adduct

DNA adducts have been studied in the mechanism of action as a carcinogen. Until 1991 [32], DNA adducts were studied in OTA-induced genotoxicity. OTA-induced adducts have been found in mice, rats, pigs and humans [32,33,34,35,36,37,38]. Manderville et al. have summarized three pathways of OTA-induced DNA adduct formation [39]. The existence of DNA adducts induced by OTA was first found using the ^32^P-post-labeling method. In the kidney, liver and spleen, DNA adducts were found after OTA treatment. However, some OTA-DNA adducts disappeared in the later stage in the 16-day experiment. The formation of OTA-DNA adducts has an obvious time and tissue dependence [32,35]. These adducts in the kidney were also found in Vero cells in 1995 [33]. However, there were more adducts in the liver of female offspring [40]. Additionally, OTA-DNA adducts were found in tumorous tissues from three kidneys and five bladders of Bulgarian patients. They mainly existed in kidney but also in bladder tissues. In 2010, [41], the structural data for the principal adduct from the OTA/DNA interaction in vitro was provided. However, designating OTA-DNA adducts as the carcinogenic mechanism of OTA is controversial. In 2004 and 2005 [42,43], Angela Mally et al. reported sequentially that no covalent DNA adducts were formed in F344 rats treated with OTA. In vitro, no postulated OTA-dG adduct was detected. Therefore, more evidence is needed to obtain the exact conclusion regarding the existence of OTA-DNA adducts.

### 2.6. Protein Synthesis Inhibition

Protein synthesis inhibition is also one of the main mechanisms induced by OTA. Protein synthesis inhibition can induce the stopping or slowing of cell growth or proliferation. The inhibition can interfere with normal cell metabolism. OTA-induced protein synthesis inhibition has been studied [44,45,46]. Creppy et al. found that the inhibition of protein synthesis induced by OTA demonstrated the inhibition of aminoacyl-tRNA synthetase, valyl-tRNA synthetase, and phenylalanyl-tRNA synthetase expression. They also found that this inhibition was found in the spleen, kidney and liver [47]. This direct evidence revealed that OTA induced the inhibition of protein synthesis.

## 3. Advances in the Epigenetic Mechanism of Ochratoxin A–Induced Toxicity

Epigenetic mechanisms are essential for the normal development and maintenance of tissue-specific gene expression patterns in mammals. Epigenetics is the modification of the activation of certain genes but not the genetic code sequence of DNA. Covalent modifications of either DNA or of histone proteins play central roles in many types of epigenetic inheritance. These modifications are mainly accomplished through two mechanisms: DNA methylation and translational modification. These disruptions of the epigenetic process may lead to altered gene function and malignant cellular transformation.

### 3.1. Effects of OTA on DNA Methylation

DNA methylation is one of the most common modes of DNA modification. In general, cytosine and adenine can be methylated. The methylation of cytosine is a process through the methyl group providing S-adenosylmethionine (SAM) combined with DNA cytosine 5 carbon atoms to form 5-mC along with the catalysis of DNA methyltransferase (DNMT). The rates of cytosine are different between species: 14% of cytosines are methylated in *Arabidopsis thaliana*, 4% in *Mus musculus*, 2.3% in *Escherichia coli*, 0.03% in *Drosophila*, and virtually none (<0.0002%) in yeast species [48]. DNA methylation can stably alter the expression of genes in transposable element silencing, genomic imprinting, embryonic development and the maintenance of genomic integrity. DNA methylation often occurs in three different nucleotide sequences: the symmetrical CpG sequence, the CHG sequence and the unsymmetrical CHH sequence (H represents the basis of A, T, or C).

DNA methylation has been a topic of considerable interest in the study of OTA toxicity in recent years. Changes in DNA methylation have become the marker of some diseases. Selvakumar et al. [49] showed that changes in the DNA methylation level may be a key mechanism in kidney disease. Zheng et al. [13] proved that OTA induced global DNA hypomethylation in HepG2 cells. Global DNA hypomethylation has been associated with aberrant gene expression and chromosomal instability. In BME-UV1 and MDCK cells, the global DNA methylation pattern has shown no change after OTA treatment [50]. In fact, the change in the global DNA methylation level by OTA induction is dynamic. Li et al. found that the level of global DNA hypomethylation was increased in the high-dose group (210 μg/kg·bw) in OTA-treated kidneys after four and 13 weeks. This may indicate that hypomethylation is an early event in OTA-induced nephrotoxicity. However, after 26 weeks, the alteration of global DNA hypomethylation disappeared [51]. The hypothesis emerged that hypermethylation alternately appears with hypomethylation in different anatomical regions or in different genomic loci. It may not reflect the real situation of OTA-induced changes in DNA methylation. This study directly detected the level of DNA methylation.

Some markers reflected the change in DNA methylation by detecting DNA methyltransferases (DNMTs). DNMTs can regulate DNA methylation and are involved in the maintenance of DNA methylation. In Li’s research [51], the relative expression of the mRNA of DNMT1 and DNMT3b was significantly increased after 13 weeks of OTA exposure. The relative expression of the mRNA of DNMT3a was significantly decreased. The different changes in DNMT also reflected that only detecting the global DNA methylation is sufficient to explore the role of DNA methylation in OTA-induced toxicity. The kidney, for instance, consists of many types of cells, and the levels of DNA methylation in these cells are different. Due to the heterogeneity, the changes in DNA methylation due to OTA were diverse. Thus, detecting the level of DNA methylation in target positions, such as the renal proximal tubule or a single cell, is more significant. Furthermore, very few DNA methylation events occur to explore the new mechanism for other OTA-induced toxicities.

### 3.2. Effects of OTA on Non-Coding RNA

Non-coding RNA (ncRNA) is an RNA molecule that is not translated into protein. Non-coding RNA genes include highly abundant and functionally important RNAs such as transfer RNAs (tRNAs) and ribosomal RNAs (rRNAs), as well as RNAs such as snoRNAs, microRNAs, siRNAs, snRNAs, exRNAs, piRNAs, scaRNAs and long ncRNAs. These ncRNAs are involved in many cellular processes. Until now, ncRNAs were mostly involved in the regulation of information flow from DNA to protein.

MicroRNA (miRNA) is a type of non-coding RNA. In the study of OTA, miRNA was explored. miRNA is a type of endogenous, conserved and single-strand RNA. miRNAs are 20 to 25 nucleotides derived from 70 to 100-base-pair hairpin-shaped precursors. It works as the regulator of gene expression in a wide range of processes via the post-transcriptional regulation of mRNA translation and stability, including the induction or maintenance of cell fate in normal, stem and cancerous cells. In a diverse range of diseases, miRNA has been investigated as a biomarker. Additionally, miRNA profiling and special miRNA have been studied in OTA-induced toxicities in vivo and in vitro.

In vivo, miRNA profiling was detected in OTA-induced nephrotoxicity and hepatotoxicity. Dai et al. [52] analyzed the miRNA profiling of the kidney. Rats were divided into different groups and underwent gavage with OTA for two, four, 13 and 26 weeks. The doses of OTA were 0 (CK), 70 (CM), and 210 ng/kg·bw (CH). Total RNA was detected in the three groups based on doses. In CK, CM and CH kidneys, 409 known miRNAs were found. Additionally, 394 miRNAs were different. After OTA treatment, the expression of *Drosha* and *Dicer* was reduced. This proved that OTA affected the integrity of the miRNA processing mechanism. In addition, there were 77 miRNAs repressed in CM and reversed in the CH group. Through KEGG/GO analysis, “phosphatidylinositol signaling system”, “pancreatic cancer” and “MAPK signaling pathway” were greatly enriched. Moreover, eight novel miRNAs were identified in this research. The research is the first to explore the toxic mechanism of OTA miRNA profiling.

In 2014, Qi et al. [53] explored the miRNA profiling in OTA-induced hepatotoxicity. In miRNA profiling, “pathways in cancer”, “MAPK signaling pathway” and “metabolic pathways” were significantly enriched in OTA-treated groups. Moreover, mRNA profiling was also detected. The results revealed that only one gene was differentially expressed in the high-dose compared with the medial-dose group. “Amino acid metabolism”, “xenobiotics biodegradation and metabolism”, “energy metabolism”, and “environmental information processing” were enriched. More importantly, seven pathways, including amino acid metabolism, lipid metabolism, signaling molecules and interaction, and xenobiotics biodegradation and metabolism, were commonly identified in the high-dose and medial-dose groups. Combined with the protein profiling, the five most relevant pathways induced by OTA, including cysteine and methionine metabolism, PPAR signaling, primary bile acid biosynthesis, arginine and proline metabolism, and metabolism of xenobiotics by cytochrome P450, are summarized.

In vitro, Zhao et al. [54] researched the cytotoxicity of OTA by using HEK293 and HepG2 as experimental models via miRNA profiling. After OTA treatment, 12 novel miRNAs were found from both miRNA profiles of HEK293 and HepG2. Eight pathways were overlapped in both cell lines from the downregulated data of miRNA profilings. They are, respectively, “pathway in cancer”, “endocytosis”, “axon guidance”, “gioma”, “postate cancer”, “MAPK signaling pathway”, “chronic myeloid leukemia” and “neurotrophin signaling pathway”. The mRNA expression levels of *Dicer*, *Drosha*, and *DGCR8* were decreased after OTA treatment. Moreover, by analyzing the miRNA profiles of the two cell lines, the common pathways were found to be mostly related to signal transduction pathways, and the different pathways were mostly related to human cancer pathways.

Based on these results in vivo and vitro, the integrity of the miRNA processing mechanism was damaged by OTA. The MAPK signaling pathway was found in all the profiles in vivo and in vitro, indicating that this pathway may be involved mainly in the toxic mechanism of OTA. In addition, novel miRNAs were identified after OTA treatment, also providing evidence that some miRNAs can be activated under special external environment stimulation.

In HEK293 cells [55], the *miR-29* family consists of *miR-29a–miR-29c*. The *miR-29* family is supposed to target at least 16 different extracellular matrices. The function of the *miR-29* family was regarded as the modulator of fibrotic processes. The study found that OTA exposure of HEK293 cells led to an increase in collagen I, III and IV protein amounts without changes in collagen mRNA expression levels; the levels were regulated by post-transcriptionally mediated mechanisms. Additionally, OTA induced an altered intracellular distribution of *miR-29b.* By contrast, adding *miR-29b* (*miR-29b* clamp) completely prevented OTA-induced collagen formation. These results revealed that OTA may initiate or support the development of fibrotic kidney diseases by involving post-transcriptional regulation mechanisms comprising *miR-29b*. Additionally, *miR-29b* may be the biomarker of fibrotic kidney diseases.

In porcine renal proximal tubular cells [17], *miR-132* and *miR-200c* were studied. Inhibition of *miR-132* restored the OTA-driven reduction in *Nrf2* expression. Moreover, *anti-miR-132* and *anti-miR-200c* counteracted the OTA-mediated decrease in HO-1 levels as well as the increase in ROS production and TGFβ expression. This also explained that OTA may induce oxidative stress by regulating the expression of miRNAs.

In the liver, *miR-122* accounts for approximately 70% of all miRNAs. *miR-122* plays a key role in normal and diseased livers. Nassirpour et al. [56] reported that *miR-122* is involved in cancer cell metabolism, apoptosis, and transfer. Moreover, *miR-122* is considered a tumor suppressor. Zhu et al. [57] studied the role of *miR-122* in OTA-induced hepatocyte apoptosis in vivo and in vitro*.* In summary, OTA induced hepatocyte apoptosis through the *CCNG1/p53* and *Bcl-w/Caspase-3* pathways via the modulation of *miR-122*. Consistent with this report, Chen et al. [58] also found that OTA can induce GC-2 cell apoptosis by causing an increase in *caspase-3* activity.

### 3.3. Effects of OTA on Histone Modification

Histone is the chief protein component of chromatin. It is an alkaline protein in cell nuclei. It can act as spools around DNA and plays a role in gene regulation. Five groups of histones exist: H1/H5, H2A, H2B, H3, and H4 [59]. Histones H2A, H2B, H3 and H4 are known as the core histones, while histones H1 and H5 are known as linker histones. The functions of histones are compacting DNA strands and chromatin regulation. Post-translational modifications can alter the histone interaction between DNA and nuclear proteins. These modifications include methylation, acetylation, phosphorylation, ubiquitination, SUMOylation, citrullination, and ADP-ribosylation. Histone modifications act in diverse biological processes such as gene regulation, DNA repair, chromosome condensation (mitosis) and spermatogenesis (meiosis) [60]. Although a huge catalog of histone modifications have been described, the functional understanding of most is still not clear.

In 2011 [61], evidence proved that histone acetyltransferases are involved in the regulation of OTA toxicity and carcinogenicity. They clearly confirmed that OTA causes sustained mitotic arrest and exit from mitosis without nuclear or cellular division. Moreover, the aberrant condensation of mitotic chromosomes and sister chromatid separation are associated with the alterations of phosphorylation and acetylation of core histones. They observed the loss of histone H3 phosphorylation at threonine 3, and OTA showed no significant direct inhibitory effect on Haspin kinase, which regulates the recruitment of the chromosomal passenger complex (CPC) to chromosomes via the phosphorylation of histone H3 at threonine-3 during mitosis. By contrast, OTA significantly blocked the histone acetyltransferase (HAT) activity of a nuclear cell extract in a concentration-dependent manner. HAT can regulate the acetylation of histones and non-histone proteins at lysine residues. These results suggested that OTA may act as an inhibitor of HAT. However, reports concerning the effect of OTA toxicity on histone modification are few. Hence, the potential toxicological mechanism of OTA induced by histone modification should be further studied.

## 4. Conclusions and Future Perspectives

**Figure 1:**
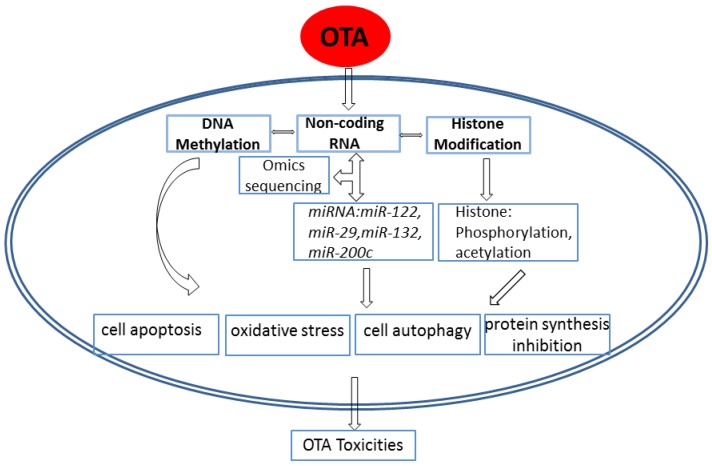
Epigenetic mechanism of Ochratoxin A–induced toxicity.

Because it establishes a mode of cancer epigenetic research, mycotoxin epigenetic research should also be explored. Overall, the results of these previous studies support the viewpoint that OTA toxicity is mainly performed through changes in epigenetics, as shown in Figure 1. The mode of OTA epigenetic research has been advanced in research hotspots. Epigenetic mechanisms of OTA-induced toxicity have been performed almost exclusively in the kidney and liver and no other organs. Moreover, these studies all chose the whole kidney or liver as the model, which cannot reveal the real changes in DNA methylation or miRNAs or histone at the target sites of OTA. More accurate alterations of epigenetic mechanisms of OTA-induced toxicity should be explored.

Recent advancements in the rapidly evolving field of mycotoxin epigenetics have shown extensive reprogramming of every component of the epigenetic machinery. However, the overall level of the research is limited. Based on these observations, the following recommendations are proposed:

(1) The specific target site of OTA should be detected by advanced technologies such as single cell sequencing, smart sequencing, reduced representation bisulfite sequencing (RRBS), and small RNA sequencing. These will provide more significant data for the understanding of OTA MOA.
(1) ceRNA can be explored with OTA. Recently, the ceRNA hypothesis was proposed. mRNA and lncRNA or circRNA may bind competitively to miRNA to regulate the expression. This may provide a new field for us to understand the OTA MOA.
